# Paroxysmal Sympathetic Hyperactivity in Severe Anti-N-Methyl-d-Aspartate Receptor Encephalitis: A Single Center Retrospective Observational Study

**DOI:** 10.3389/fimmu.2021.665183

**Published:** 2021-04-12

**Authors:** Dongmei Wang, Shuang Su, Miaoqin Tan, Yongming Wu, Shengnan Wang

**Affiliations:** ^1^ Department of Neurology, Nanfang Hospital, Southern Medical University, Guangzhou, China; ^2^ Department of Geriatrics, Nanfang Hospital, Southern Medical University, Guangzhou, China

**Keywords:** anti-NMDA receptor encephalitis, paroxysmal sympathetic hyperactivity, autonomic dysfunction, neuro-intensive care unit, treatment

## Abstract

**Background:**

Paroxysmal sympathetic hyperactivity (PSH) is a disorder with excessive sympathetic activity commonly recognized in patients with acquired brain injury. Autonomic instability is frequent in anti-N-methyl-d-aspartate receptor encephalitis (anti-NMDARE). However, PSH in anti-NMDARE has gained little attention.

**Methods:**

We retrospectively reviewed 24 patients diagnosed with severe anti-NMDARE in the neuro-intensive care unit (NICU) between 2014 and 2019. Patients were assessed with the PSH assessment measure (PSH-AM) scale, and categorized into “PSH+” group and “PSH-” group. The clinical characteristics, hospital mortality, and functional outcome by modified Rankin Scale (mRS) score at six months after discharge were compared between the two groups. Among patients with PSH+, the clinical features and pharmacotherapy of PSH were summarized and compared.

**Results:**

Twenty-four patients were included in the study. Twelve of them (50%) were categorized as PSH+ based on PSH-AM scores. There were no significant differences in the demographic characteristic, GCS scores upon admission, incidence of status epilepticus, teratoma occurrence, hospital mortality, and 6-month mRS between PSH+ and PSH- groups. Patients with PSH+ had increased length of NICU stay, hospital stay and duration of mechanical ventilation. The most prominent clinical features of PSH in severe anti-NMDARE were tachycardia and hyperthermia, while posturing was the relatively mildest clinical feature. Propranolol and clonazepam were more commonly used than gabapentin in pharmacotherapy of PSH in severe anti-NMDARE.

**Conclusions:**

The incidence of PSH in severe anti-NMDARE patients was as high as 50%. Patients with PSH demonstrated prolonged NICU stay, hospital stay and increased duration of mechanical ventilation, while no effect on hospital mortality and functional outcome. Clinicians should be aware of the distinctive characteristics and treatment options of PSH in severe anti-NMDARE.

## Introduction

Paroxysmal sympathetic hyperactivity (PSH) is a hyper-adrenergic clinical syndrome, characterized by episodic tachycardia, hypertension, tachypnea, hyperpyrexia, diaphoresis and abnormal motor posturing (1). It is most frequently recognized in traumatic brain injury (TBI), as well as anoxic brain injury and stroke etc. The pathogenesis of PSH is unclear, but it is generally believed to be associated with the sympathetic balance model proposed by Baguley (2), the excitatory: inhibitory ratio model (EIR). In EIR model, the absence of descending inhibition results in exaggerated spinal reactivity, with sympathetic efflux triggered by non-nociceptive peripheral stimuli. Recognition of PSH is important, because it’s not rare in severe brain injury, associated with increased mortality, higher healthcare costs, longer hospitalizations, and poorer outcomes (3, 4).

Anti-N-methyl-d-aspartate receptor (anti-NMDAR) encephalitis is the most common autoimmune encephalitis (5), characterized by six main symptoms, including psychiatric symptoms or cognition impairment, seizure, speech dysfunction, movement disorder, impaired consciousness, and autonomic dysfunction or central hypoventilation, commonly requiring neuro-intensive care unit (NICU) support (5). Autonomic dysfunction is frequent in anti-NMDAR encephalitis (anti-NMDARE), including central hyperthermia/hypothermia, arrhythmia (sinus tachycardia/sinus bradycardia), abnormal blood pressure (hypertension/hypotension), central hypoventilation/hyperventilation, sexual dysfunction (erectile dysfunction), hyperhidrosis, hypersalivation and urinary incontinence (6). There have been some individual case reports about anti-NMDARE with PSH (7, 8). However, few reports elucidate the incidence, clinical manifestation, treatment and prognosis of anti-NMDARE patients with PSH.

In this study, we performed a retrospective observational cohort study in patients with severe anti-NMDARE, using the PSH-AM scale, aiming to illustrate the characteristics of PSH in severe anti-NMDARE.

## Methods

### Study Design and Participants

We screened all patients with a diagnosis of anti-NMDARE admitted to the NICU of Nanfang Hospital, a tertiary university-affiliated academic hospital in south China, from 2014 to 2019. The criteria of a diagnosis of anti-NMDARE were as follows (9): rapid onset (less than 3 months) of one or more of the six major groups of symptoms, including abnormal (psychiatric) behavior or cognitive dysfunction, speech dysfunction (pressured speech, verbal reduction, and mutism), seizures, movement disorders (dyskinesias or rigidity/abnormal postures), decreased level of consciousness, and autonomic dysfunction or central hypoventilation; the presence of NMDAR antibody; exclusion of other diseases.

Severe anti-NMDARE was defined as anti-NMDARE fulfilling one or more of following criteria (5): respiratory failure requiring endotracheal intubation and/or mechanical ventilation; disturbance of consciousness; status epilepticus. The exclusion criteria of the study were listed as following (10, 11): accompanied with other encephalitis; history of hypertension; renal failure; cardiomyopathy; brain tumor; pheochromocytoma; data missing.

The diagnostic criteria of PSH were based on the PSH-AM scale, consisting of the Clinical Feature Scale (CFS) and the Diagnosis Likelihood Tool (DLT) (1). CFS is to assess the severity of the six clinical features of PSH (heart rate, respiratory rate, systolic blood pressure, temperature, sweating and posturing). DLT is to address the diagnostic specificity, consisting of 11 diagnostic features. The PSH-AM score is achieved by adding the CFS and DLT scores together, with < 8 as unlikely, 8–16 as possible, and ≥17 as probable (1). Patients were primarily scored based on the electrical medical records by 2 individual neurologists. The patient was defined as “PSH+” if he/she had a PSH-AM score ≥ 8, otherwise as “PSH-”.

### Data Collection

Electrical medical records were carefully reviewed to collect the patients’ information. Demographic, Glasgow coma scale (GCS) scores on admission, six clinical characteristics of PSH, max PSH-AM scores during hospitalization, teratoma, status epilepticus (SE), length of NICU stay, hospital stay, duration of mechanical ventilation (MV), intervals between disease onset and PSH onset, medication used for PSH, in-hospital mortality, and neurological outcome by 6-month modified Rankin Scale (mRS) were retrospectively collected. Neurological outcome was evaluated by telephone interviews with a trained neuroscientist blinded to study design or by the clinical records if he/she was followed-up in our clinic.

This study was approved by the Ethics Committee of Nanfang Hospital, Southern Medical University. Informed consent was waived by the review board because this study was observational and retrospective, and all data were fully de-identified.

### Statistical Analysis

Student’s t-test was used to compare continuous variables. Mann-Whitney and Chi-Squared tests were used to compare non-continuous and categorical variables between the two groups. Statistically significant was defined as p-value < 0.05. SPSS statistical software v.22 was used for the statistical analysis.

## Results

**Figure 1:**
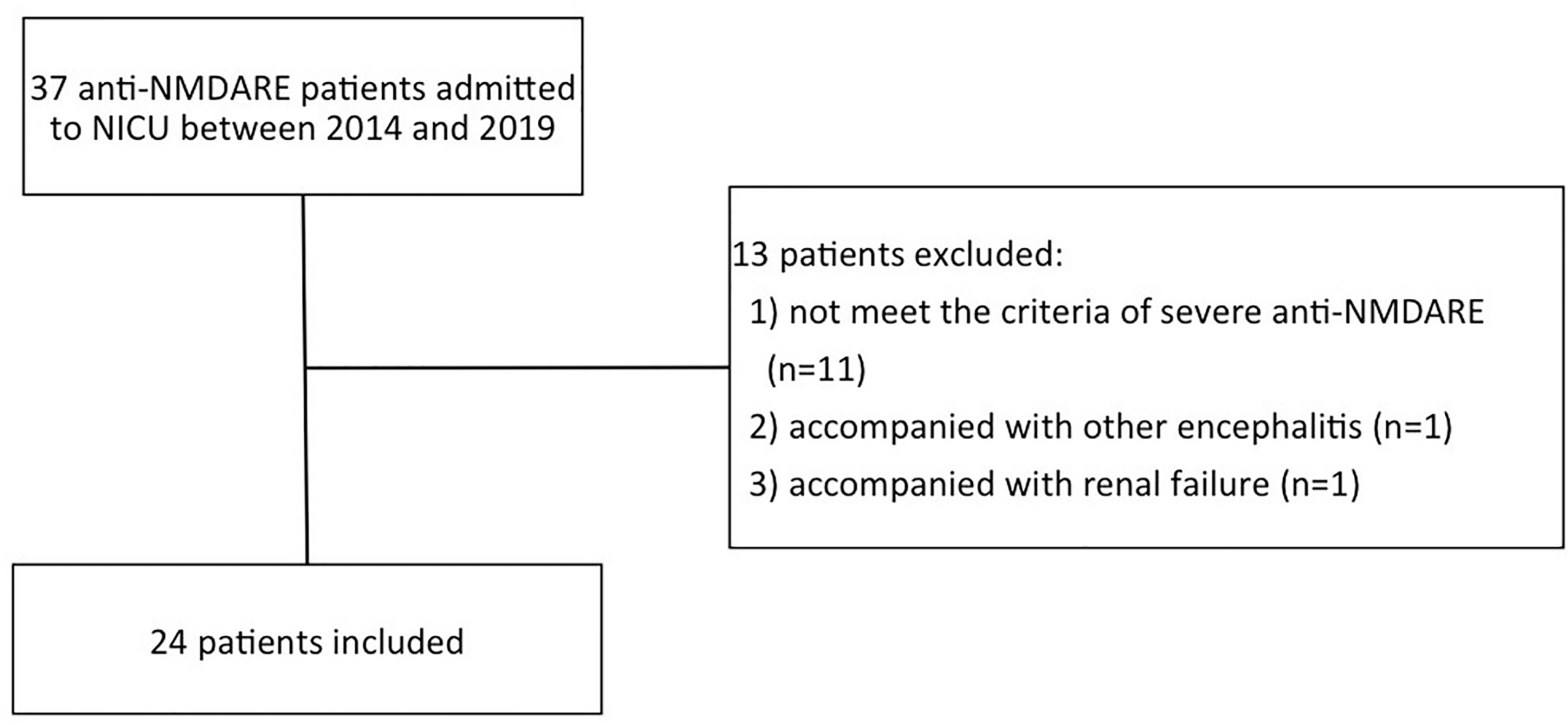
The patient selection flowchart. anti-NMDARE: anti-N-methyl-d-aspartate receptor encephalitis; NICU, neuro-intensive care unit.

Of 37 anti-NMDARE patients, 24 patients were finally included in **Figure 1**. In this cohort, 12 patients (50%) were categorized as PSH+, while 12 categorized as PSH-. The comparisons of clinical characteristics between the PSH+ group and the PSH- group were shown in **Table 1**. There were no significant differences between the two groups in demographic characteristic, GCS s2cores upon admission, incidence of SE and teratoma, hospital mortality, and 6-month mRS score. All the patients underwent the first-line therapies, including teratoma resection if exists, steroids, intravenous-immunoglobulin (IVIG), and plasma-exchange (PE). One patient with PSH+ was excluded from the analysis of NICU and hospital stay to avoid bias, due to the extremely long NICU and hospital stay (hospital stay of 666 days, NICU stay of 511 days) (12). After adjustment, compared with PSH- group, patients in PSH+ group had significantly higher incidence of MV (P=0.039), increased duration of MV (P=0.028), longer NICU (P=0.019) and hospital stay (P=0.019).

**Table 1:** Baseline characteristics of the two groups.

Variables	PSH+(n = 12)	PSH-(n = 12)	*P*
**Gender (female), n (%)**	9 (75%)	10 (83.3%)	1.000^b^
**Age, mean ± SD, y**	24.67 ± 6.48	30.33 ± 12.15	0.084^c^
**GCS, median [IQR]**	7 (4,8)	10.5 (3,12)	0.410^a^
**mechanical ventilation (MV) n (%)**	9 (75%)	3 (25%)	**0.039^b^**
**Duration of MV, median [IQR]**	8 (0.5,31)	0 (0,0.75)	**0.028^a^**
**SE, n (%)**	8 (6.7%)	4 (33.3%)	0.220^b^
**Teratoma, n (%)**	5 (41.7%)	4 (33.3%)	1.000^b^
**First-line therapy, n (%)**			
**Teratoma resection**	5 (41.7%)	4 (33.3%)	1.000^b^
**Steroids**	9 (75%)	5 (41.7%)	0.214^b^
**IVIG**	11 (91.7%)	12 (100%)	1.000^b^
**PE**	11 (91.7%)	7 (58.3%)	0.155^b^
**Days after onset of anti-NMDARE, median [IQR]**	28 (18.75, 38.75)	NA	NA
*length of NICU stay, median [IQR]	52 (30,96)	12.5 (5,41)	**0.019^a^**
*Hospital stay, median [IQR]	80 (39,96)	30 (20,45.75)	**0.019^a^**
**Hospital mortality, n (%)**	0 (0.0%)	1 (8.3%)	1.000^b^
**6-month mRS, n (%)**			
**0-2**	9 (75%)	9 (81.8%)	0.619^b^
**3-5**	1 (8.3%)	0 (0.0%)	
**6**	2 (16.7%)	2 (18.2%)	

^a^Mann-Whitney U test; ^b^χ^2^ test; ^c^Student’s t-test.

*exclude one patient for her extremely long hospital stay and NICU stay.

GCS, Glasgow coma scale; IVIG, intravenous immunoglobin; mRS, modified Rankin Scale; NA, not available; NICU, neuro-intensive care unit; PE, plasma exchange; PSH, paroxysmal sympathetic hyperactivity; SE, status epilepticus.

Bold: statistically significant.

**Table 2** shows the clinical characteristics of the 12 patients with PSH. The median onset of PSH occurred 28 days after the onset of anti-NMDARE. According to CFS, the mean scores of six symptoms in PSH+ patients were 2.67 ± 0.49 (heart rate), 2.33 ± 0.89 (respiratory rate), 2.00 ± 0.74 (systolic blood pressure), 2.67 ± 0.49 (temperature), 1.33 ± 0.65 (sweating) and 1.17 ± 0.12 (posturing), respectively. The most prominent clinical features of PSH in severe anti-NMDARE were tachycardia and hyperthermia, followed by tachypnea, hypertension, and dystonia. Posturing was relatively milder than other five symptoms. The mean CFS scores was 12.00 ± 3.41 in PSH+ group, suggesting PSH in severe anti-NMDARE was moderate to severe (1). In addition, 2 patients in PSH+ group also complicated with bradycardia and asystole.

**Table 2:** The CFS score of PSH in PSH (+) group.

PSH presentation	CFS (mean ± SD)
Heart rate	2.67 ± 0.49
Respiratory rate	2.33 ± 0.89
Systolic blood pressure	2.00 ± 0.74
Temperature	2.67 ± 0.49
Sweating	1.33 ± 0.65
Posturing	1.17 ± 0.12
Total	12.0 ± 3.41

The specific clinical features of PSH (heart rate, respiratory rate, systolic blood pressure, temperature, sweating and posturing) were examined and assigned 0 to 3 points (0 = nil; 1=mild; 2 = moderate; 3 = severe) to assess the severity of the clinical features of PSH. The total of the CFS for the six features was used to determine a severity score (0 = nil; 1–6=mild; 7–12 = moderate; ≥13 = severe). CFS, clinical feature scale; PSH, paroxysmal sympathetic hyperactivity.

**Figure 2:**
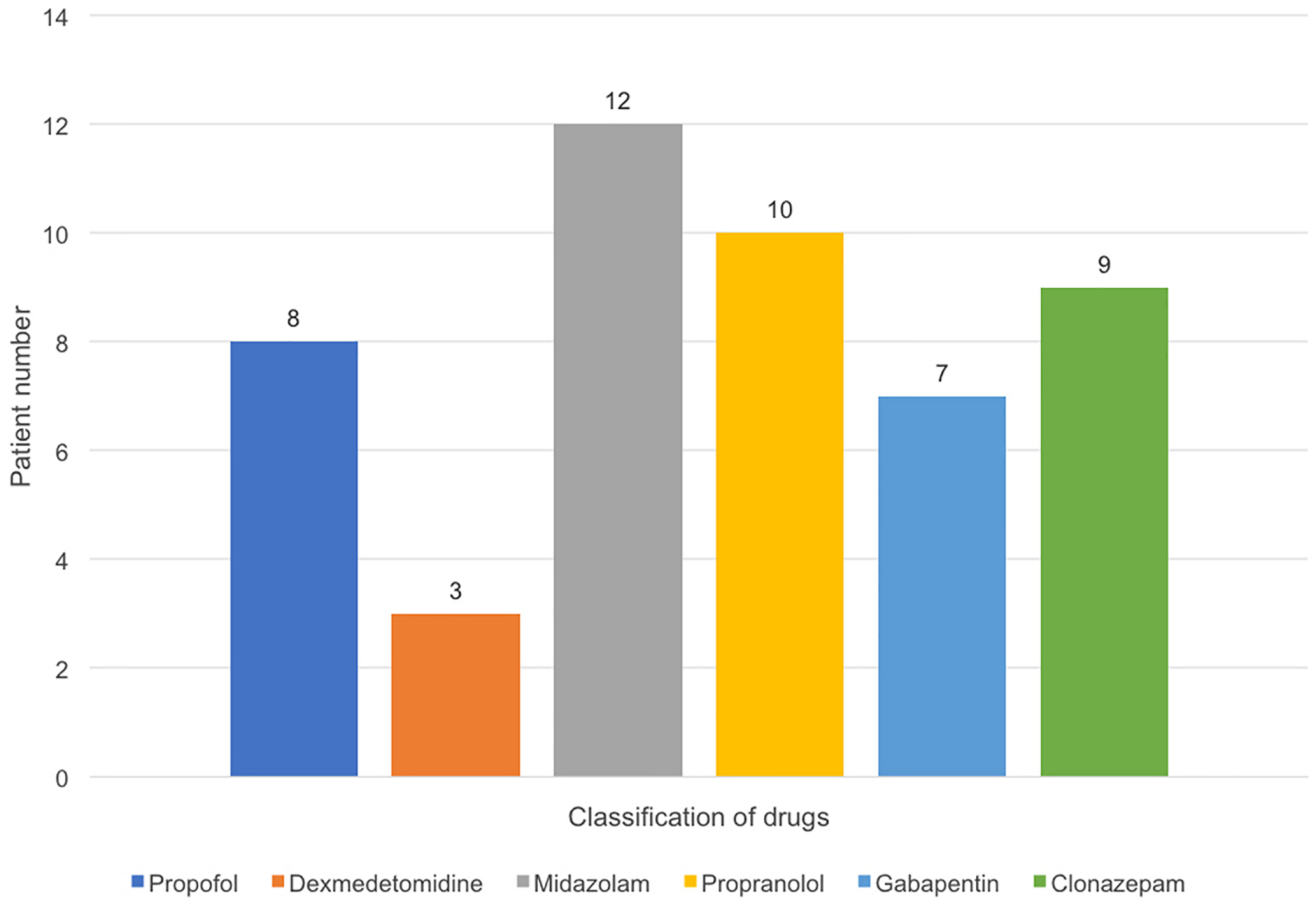
Pharmacologic management in PSH (+) group. The most common intravenous treatment for acute symptom termination was midazol am (n = 12), followed by propofol (n = 8), and dexmedetomidine (n = 3). For chronic prevention, propranolol (n = 10) was the most common option, followed by clonazepam (n = 9) and gabapentin (n = 7).

Medications used for treatment and prevention of PSH were shown in **Figure 2**. The most common intravenous treatment for acute symptom termination was midazolam (n=12, 100%), followed by propofol (n=8, 66.6%), and dexmedetomidine (n=3, 25%). For chronic prevention, propranolol (n=10, 83.3%) was the most common option, followed by clonazepam (n=9, 75%) and gabapentin (n=7, 58.3%).

## Discussion

We performed a retrospective observational cohort study and found that the incidence of PSH in severe NMDARE was as high as 50%. The most remarkable clinical manifestations of PSH in severe NMDARE were tachycardia and hyperthermia. PSH would appear days to months after the onset of anti-NMDARE, and be associated with longer NICU day and hospital stay, and increased duration of MV. However, PSH had no significant effect on mortality and functional outcome.

Autonomic instability is a frequent feature of anti-NMDARE (9), with the underlying mechanism remaining unclear. It is generally believed to be associated with the NMDAR hypofunction, which affects the sympathetic and parasympathetic nervous systems (9, 13). In this theory, NMDAR antibodies decrease NMDAR surface density and synaptic localization *via* selective antibody-mediated capping and internalization of surface NMDARs, which abolishes NMDAR-mediated synaptic currents, affecting the dopaminergic, adrenergic and cholinergic systems. As reported, the non-competitive NMDAR antagonists (phencyclidine and ketamine) can induce similar autonomic dysfunction symptoms as in anti-NMDARE, while NMDAR agonists can improve these symptoms (9). According to the diagnostic criteria of PSH, there is overlap between the autonomic instability and PSH. There have been some individual case reports about anti-NMDARE with PSH (7, 8). In our study, we found the incidence of PSH in severe NMDARE was 50%, while no risk factors were identified. In previous literature about brain injuries, PSH is more common in males (10), younger patients (14–16), and patients with lower GCS scores (4, 11). However, no significant risk factors were found in this study, although patients with PSH tended to be younger (P=0.084). This result may be due to the small sample size and clinical characteristics of the selected patients, because severe anti-NMDARE favored young females and low GCS scores. A larger study was needed to elucidate the risk factors.

Tachycardia, tachypnoea, hypertension, hyperthermia, sweating, and posturing are not simultaneously and equally occurred in all the patients with PSH (1, 17). More autonomic features would increase diagnostic sensitivity and specificity (16). In our study, we found that tachycardia and hyperthermia were the most significant clinical manifestations of PSH in severe anti-NMDARE. The result was consistent with a previous observational cohort study by Hinson et al. (18), which reported hyperthermia was the most predictive component of PSH by an objective record. Besides, we found posturing relatively milder than other autonomic features. These clinical manifestations led to the specified choice of chronic pharmacotherapy for PSH in our study: propranolol and clonazepam were more commonly used than gabapentin. The main purpose of managing PSH is to avoid PSH triggers, to block the excessive sympathetic hyperactivity and to reduce the incidence of complication. The most frequently used medications of PSH are opioids and β-blockers, with first-line therapy of benzodiazepines, centrally acting α-agonists, and gabapentin as well (19). It is generalized believed that nonselective β-blockers target hypertension, tachycardia, and hyperthermia; benzodiazepines target hypertension, tachycardia, and posturing; while gabapentin targets spasticity. Although propranolol is the main treatment for PSH, it should be emphasized that the autonomic dysfunction in anti-NMDARE includes the activation of both sympathetic and parasympathetic nervous systems, and bradycardia or asystole is not uncommon (20, 21). Some severe patients may even require transvenous temporary cardiac pacing and permanent cardiac pacemaker implantation due to severe bradyarrhythmia (21). Thus, β-blockers should be used with caution. Gabapentin is more effective for reducing spasticity, which was less common and milder in our study. Therefore, appropriate medication for PSH should be carefully selected according to the specific clinical manifestations.

PSH was associated with longer NICU and hospital stay, prolonged MV, instead of mortality and functional outcome in this study. Currently, the effect of PSH on outcome remains controversial. In some studies, the occurrence of PSH was mirrored by a poorer prognosis (22), while others did not find significant differences than matched controls (15). In a review of 10 literatures (3), five studies reported that PSH was associated with longer NICU and hospital stay (23–26), while two had no significant differences (17, 27), and the other three had no assessment (17, 28). Some studies showed longer duration of MV (24, 28), while one had no significant difference (26), others no assessment. In addition, four studies reported PSH was associated with a poorer prognosis (17, 23, 25, 26), while five found no significant differences. Geert et al. (3) find outcomes of PSH might depend on disease duration, pharmacologic interventions, clinical complications and the primary disease. Despite these uncertainties, PSH is an independent risk factor for poor neurological outcomes in the overall clinical impression (3). In our study, no significant differences in hospital mortality and 6-month outcome were observed between PSH+ and PSH- groups, which might be associated with the active therapy of anti-NMDARE and progressive management of complications. Longer NICU and hospital stay, prolonged MV could be caused by the clinical complications and the differential diagnoses provoked by PSH, such as secondary brain damage, cardiac arrhythmias and fever of unknown origin. In addition, PSH is frequently associated with more severe brain injury (1, 3, 11, 16, 17), which means a longer NICU and hospital stay, and prolonged MV.

This study has several limitations on the retrospective observation design and relatively small sample size. However, this is the first study focusing on PSH in severe anti-NMDARE, which suggested that the incidence of PSH was as high as 50%. Clinicians should be aware of PSH and pay attention to the management of PSH. A large study is warranted to confirm these results.

## Conclusions

In this study, we found that the incidence of PSH in severe NMDARE was 50%. The most remarkable clinical manifestation of PSH in severe NMDARE was tachycardia and hyperthermia. PSH was associated with longer NICU day, hospital stay, and duration of mechanical ventilation. However, it had no effect on mortality and functional outcome.

## Data Availability Statement

The raw data supporting the conclusions of this article will be made available by the authors, without undue reservation.

## Ethics Statement 

This study was approved by the Ethics Committee of Nanfang Hospital, Southern Medical University. Informed consent was waived by the review board because this study was observational and retrospective, and all data were fully de-identified.

## Author Contributions

DW, YW, and SW are responsible for concepts and design. MT and SS are responsible for data collecting and statistical analysis. All authors contributed to the article and approved the submitted version. All authors acquired, analyzed, and interpreted the data. The manuscript was prepared by DW and SS.

## Funding

This study was supported by President Foundation of Nanfang Hospital, Southern Medical University (No.2020B006).

## Conflict of Interest

The authors declare that the research was conducted in the absence of any commercial or financial relationships that could be construed as a potential conflict of interest.
